# Visual Cortical Function Changes After Perceptual Learning with Dichoptic Attention Tasks in Adults with Amblyopia: A Case Study Evaluated Using fMRI

**DOI:** 10.3390/brainsci14111148

**Published:** 2024-11-16

**Authors:** Chuan Hou, Zhangziyi Zhou, Ismet Joan Uner, Spero C. Nicholas

**Affiliations:** The Smith-Kettlewell Eye Research Institute, San Francisco, CA 94115, USA; zhangziyi.zhou@ski.org (Z.Z.); ismet.uner@gmail.com (I.J.U.); speronicholas@gmail.com (S.C.N.)

**Keywords:** amblyopia, strabismus, perceptual learning, visual cortex, fMRI, visual attention

## Abstract

**Background:** Amblyopia is a neurodevelopmental disorder of vision, commonly caused by strabismus or anisometropia during early childhood. While studies demonstrated that perceptual learning improves visual acuity and stereopsis in adults with amblyopia, accompanying changes in visual cortical function remain unclear. **Methods:** We measured functional magnetic resonance imaging (fMRI) responses before and after perceptual learning in seven adults with amblyopia. Our learning tasks involved dichoptic high-attention-demand tasks that avoided V1 function-related tasks and required high-level cortical functions (e.g., intraparietal sulcus) to train the amblyopic eye. **Results:** Perceptual learning induced low-level visual cortical function changes, which were strongly associated with the etiology of amblyopia and visual function improvements. Anisometropic amblyopes showed functional improvements across all regions of interest (ROIs: V1, V2, V3, V3A, and hV4), along with improvements in visual acuity and stereoacuity. In contrast, strabismic amblyopes showed robust improvements in visual cortical functions only in individuals who experienced significant gains in visual acuity and stereoacuity. Notably, improvements in V1 functions were significantly correlated with the magnitude of visual acuity and stereoacuity improvements when combining both anisometropic and strabismic amblyopes. **Conclusions:** Our findings provide evidence that learning occurs in both high-level and low-level cortical processes. Our study suggests that early intervention to correct eye alignment (e.g., strabismus surgery) is critical for restoring both visual and cortical functions in strabismic amblyopia.

## 1. Introduction

Amblyopia, or “lazy eye”, is a neurodevelopmental disorder of vision and affects about 3% to 5% of the population [1]. The standard clinical treatments are best optical correction and patching the fellow eye (occlusion therapy) in children and teenagers. Traditionally, amblyopia was considered largely untreatable in adults due to reduced neural plasticity [2,3]. However, in recent decades, studies [4,5,6,7,8,9,10,11,12,13] have shown that perceptual learning or training (repeated practice on a demanding visual task, also referred to as “training” in the current study) can lead to significant improvements in visual function in amblyopic adults, even beyond the critical developmental period for human vision. For a comprehensive review, see Levi (2005; 2009; 2009) [14,15,16], Hess et al. (2014) [17], Tsrilin et al. (2015) [18], and Rodán et al. (2022) [19]. 

Nevertheless, it remains unclear whether and how the significant visual function improvements (e.g., visual acuity and stereoacuity) in amblyopic adults through perceptual learning are accompanied by changes in visual cortical function. There is limited documentation on this topic. For instance, it remains an open question whether cortical function changes occur at a lower level (e.g., the primary visual cortex, V1), at a higher ”decision stage” of visual processing, such as the intraparietal sulcus (IPS)/prefrontal cortex, or both (e.g., via feedback at a low level but under top-down control) [20,21]. One functional magnetic resonance imaging (fMRI) study (Zhai et al., 2013) [22] reported that, following perceptual learning, increased activation in Brodmann Areas 17–19, which correspond to V1, V2, V3, and V4, was observed in patients with anisometropic amblyopia while their visual acuity improved. This study used a low-level training task, where the grating stimulus with a “cut-off” frequency was applied to train the amblyopic eye for 40 min per day over 30 days. Typically, learning effects with stimulus specificity in low-level processing tasks (e.g., spatial frequencies, contrast detection, and contrast-dependent orientation discrimination) are considered evidence that perceptual learning reflects plasticity in early visual cortical areas [23,24,25,26]. This is because local contrast encoding (edge detection), orientation selectivity, and spatial frequencies are properties of neurons in V1 [27]. Thus, Zhai et al. (2013) [22], using “cut-off” frequency tasks and the observation of increased activation in Brodmann Areas 17–19, further confirmed the principle that low-level tasks induce early visual cortex changes in anisometropic amblyopia. However, it remains unclear whether strabismic amblyopia follows the same pattern of visual cortical function recovery as anisometropic amblyopia. In this study, we included adults with both anisometropic and strabismic amblyopia for a more comprehensive comparison.

On the other hand, recent perceptual learning studies using either dichoptic video games [8,28] or monocular action video games [4,29,30] to train adults and children with amblyopia have also resulted in significant improvements in visual acuity and stereoacuity. These studies suggest that learning occurs at a higher “decision stage” of visual processing. This is because action video games enhance selective visual attention both in space (e.g., more effective localization of a target among distractors [31]) and in time (e.g., enhancing the ability to select relevant information over time [32]), which involves higher-level cortical functions, such as those in the parietal and prefrontal cortices (see Palaus et al., 2017 [33], for a review). Attention refers to the behavioral and cognitive process of selectively focusing on relevant information while ignoring irrelevant details. This process engages higher-level visual pathways and working memory (see Knudsen, 2007 [34], for a review). An open question remains: do high-level training tasks that target high-level cortical processing, such as video game tasks [35,36] or attention-based tasks [13], also induce functional changes in early visual cortex, such as V1?

Therefore, in this study, we aimed to investigate whether using perceptual learning tasks, which avoided V1 function-related tasks (e.g., contrast detection task or spatial frequency “cut-off” task) [37] but engaged high-level cortical functions (e.g., IPS) [38,39], could train the amblyopic eye to induce early visual cortical function changes in adults with anisometropic and strabismic amblyopia.

## 2. Materials and Methods

### 2.1. Subjects

Seven adults (5 females) with amblyopia (3 anisometropic and 4 with a mix of anisometropic and strabismic) participated in this study. The age range was between 28 and 66 years (mean ± SD, 49.43 ± 12.40). All participants underwent eye examinations and were refracted under a non-cycloplegic condition by one of the authors (C.H.), a pediatric ophthalmologist, before perceptual learning. Visual acuity was measured at 6 m with a logMAR chart (Bailey-Lovie) at the best optical correction. The inclusion criteria were as follows: participants with best-corrected visual acuity in the amblyopic eye (AE) of 0.20 logMAR (20/30) or worse and in the fellow eye (FE) of 0.00 logMAR (20/20) or better. Stereoacuity was evaluated with the Random-Dot Stereo Butterfly card (Stereo Optical, Chicago, IL, USA) at 40 cm with the best optical correction. Amblyopia was defined as anisometropic amblyopia (≥1 D of refractive error interocular difference, referred to as “Aniso”), strabismic amblyopia, or a mix of both. We refer to strabismic or mixed amblyopia as “Strab” in this study. Individuals with other eye conditions, e.g., cataract, glaucoma, age-related macular degeneration, or other types of amblyopia (e.g., secondary to congenital cataract) were excluded in this study. All participants were screened for the presence of monocular fixation instability and eccentric fixation using a direct ophthalmoscope. Participants who had eccentric fixation and nystagmus or latent nystagmus (nystagmus that appears when covering one eye) were excluded from the study. Clinical information is provided in Table 1. 

### 2.2. Perceptual Leaning

All subjects participated in a previously reported study on perceptual learning using dichoptic attention tasks (Hou and Nicholas, 2022) [13]. Visual function improvements, including visual acuity and stereoacuity along with reductions in interocular suppression and training task performances, were reported by Hou and Nicholas (2022). Here, in this study, we reported 7 subjects who had visual cortical function evaluation using fMRI pre- and post-perceptual learning. 

**Figure 1:**
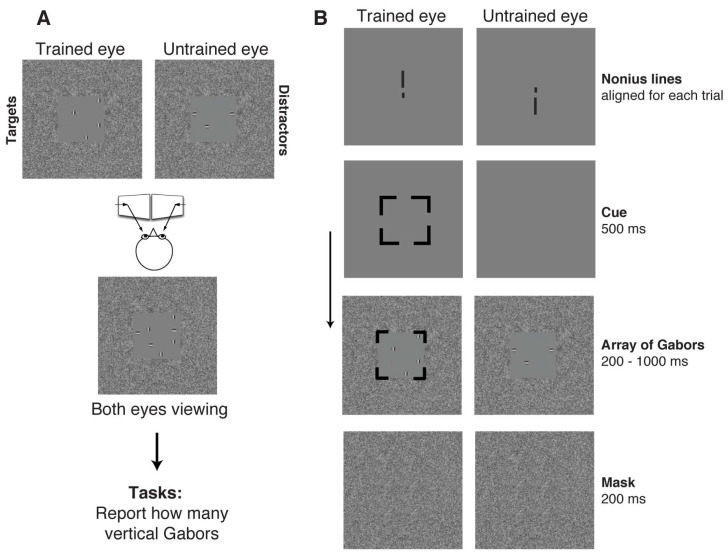
An illustration of perceptual learning stimuli adopted from Hou and Nicholas (2022) [13]. (**A**) The perception of a given trial with targets (vertical Gabors) in the trained eye and distractors (horizontal Gabors) in the untrained eye under dichoptic viewing through a mirror stereoscope. (**B**) The temporal sequence of a given trial in perceptual learning sessions. A random array of highly visible Gabor patches was presented in the trained eye with vertical Gabors (targets) and in the untrained eye with horizontal Gabors (distractors) followed by a 200 ms noise mask. A 500 ms valid attentive cue preceded each trial indicating which eye would receive the targets. The trials were self-initiated, and participants were requested to respond as accurately as possible with no time limit, and no feedback was given.

Perceptual learning tasks: Our 7 subjects were trained for 2 h each with about two visits per week for two months using high-attention-demand tasks in a dichoptic approach; see details in the study by Hou and Nicholas (2022) [13]. In brief, as shown in Figure 1, our training included three key factors (searching [40], counting [34], and attentional cueing [34]) with implemented selective visual attention in the tasks, thus incorporating “high attention demand”. The tasks consisted of quickly searching and counting “targets” presented to the amblyopic eye among “distractors” that were simultaneously presented to the fellow eye preceded by 500 ms attentional cueing to the amblyopic eye. Our learning avoided V1 function-related tasks, such as contrast detection or spatial frequency “cut-off”, and involved visual attention processes [41] as well as high-level visual pathways [41] and the IPS [38,39].

### 2.3. Structural and Functional MRI 

#### 2.3.1. Stimuli

**Figure 2:**
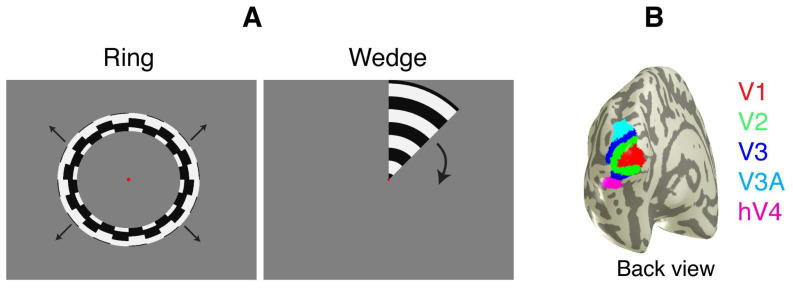
Stimuli and retinotopic regions of interest (ROIs). (**A**) An illustration of the stimuli (expanding rings and rotating wedges of contrast-reversing checkerboards) used in fMRI scans, adapted from Engel et al. (1997) [43]. Red dot indicates fixation point. Black arrows indicate direction. (**B**) ROIs corresponding to early visual cortical areas V1, V2, V3, V3A, and human V4 (hV4) in each hemisphere (demonstrated at the left hemisphere).

The expanding rings and rotating wedges of contrast-reversing checkerboards adapted from (Dougherty et al., 2003) [42] and Engel et al. (1997) [43] were used for fMRI in our study. The stimuli were created with VISTADISP (GitHub-vistalab/vistadisp). As shown in Figure 2A, the ring made of drifting alternately moved radially towards and away from fixation at a velocity of 1°/s and completed a full expansion every 24 s. The wedge spanned a 45° angle and extended to 12° from the fixation. The wedge completed a full rotation every 24 s. Both stimuli changed positions in synchrony with the data acquisition frame rate of 2 s TR for Trio scanner (Siemens Healthineers, Erlangen, Germany) and 1.5 TR for Plasma scanner (Siemens Healthineers, Erlangen, Germany). The stimuli were presented on a liquid crystal display behind the bore, viewed through a mirror at a distance of 122 cm, providing a 14° diameter field of view.

#### 2.3.2. Data Acquisition

Two MRI sessions were carried out for each subject before and after perceptual learning at the Neuroscape MRI Lab at UCSF (University of California, San Francisco) Mission Bay campus. MRI data were acquired on a 3T Siemens Tim Trio with a 12-channel head coil for 4 participants (A1, A2, S1, and S3) and on a 3T Siemens Prisma with a 64-channel head coil for 3 participants (A3, S2, and S4). (Both Prisma and Trio scanners are manufactured by Siemens Healthineers, Erlangen, Germany) We acquired a T1-weighted MRI data set (3D MP-RAGE sequence, 0.8 × 0.8 × 0.8 mm^3^) and a 3D T2-weighted data set (SE sequence at 1 × 1 × 1 mm^3^ resolution for Trio scanner and 0.8 × 0.8 × 0.8 mm^3^ for Prisma scanner) for tissue segmentation and registration with the functional scans [44]. For fMRI, on a Trio scanner, we employed a single-shot, gradient-echo planar imaging (EPI) sequence (TR/TE = 2000/28 ms, flip angle 80, 126 volumes per run) with a voxel size of 1.72 × 1.72 × 2 mm^3^ (128 × 128 acquisition matrix, 220 mm field of view, bandwidth of 1860 Hz/pixel, and echo spacing of 0.71 ms). On a Prisma scanner, we employed a single-shot, gradient-echo planar imaging (EPI) sequence (TR/TE = 1500/30 ms, flip angle of 45, and 168 volumes per run) and a voxel size of 1.6 × 1.6 × 1.6 mm^3^ (136 × 136 acquisition matrix, 220 mm field of view, bandwidth of 1934 Hz/pixel, and echo spacing of 0.64 ms). The stimuli consisted of expanding rings and rotating wedges, and each were repeated 4–5 times (variability was due to the amount of prep time for the participants to become comfortably situated in the scanner) for a scanning session under the best optical correction for each participant. A color-switching dot (red/green) was placed at the fixation point in the center of the screen to ensure that the participant was attending to the stimuli. Participants were instructed to press the button on a controller whenever the dot changes color. The total acquisition time for each subject’s MRI scan was 1.5 h. 

#### 2.3.3. Data Analysis and Visual Area Definition 

The fMRI (BOLD) response was analyzed by extracting the Fourier fundamental of the time series at every voxel at the stimulus alternation rate of 1/24 (0.0416 Hz). Since each subject had separate fMRI scans for pre- and post-training, we normalized the data in each fMRI session by computing the signal-to-noise ratio (SNR) [43], which served as a normalization method to control variability across the two sessions. We followed a similar approach as described in Engel et al. (1997), where the SNR was computed by dividing the signal (Fourier fundamental of the stimulus alternation rate at 0.0416 Hz) amplitude by the associated noise amplitude, which was defined as the average of the amplitudes from two adjacent frequency bins (0.0375 Hz and 0.0459 Hz) at the stimulus alternation rate. The standard errors were calculated using the mean values across voxels in each ROI. Each individual fMRI run contained 10.5 cycles of the rotating wedge or expanding ring stimuli lasting 24 s, where the first half-cycle was excluded from the analysis to remove the effects of the transient onset of the sequence. The FreeSurfer software package (http://surfer.nmr.mgh.harvard.edu (accessed on 10/7/2018)) was used to perform gray and white matter segmentation and to extract the midgray cortical surface. We used the boundary element method (the FMRIB Software Library; http://fsl.fmrib.ox.ac.uk/fsl/ (accessed on 10/7/2018)) with boundary surfaces derived from the T1- and T2-weighted MRI scans of each subject [45,46]. As described in previous studies [43,47], retinotopic regions of interest (ROIs) corresponding to areas in early visual cortical areas V1, V2v, V2d, V3v, V3d, V3a, and hV4 in each hemisphere (Figure 2B) were mapped using rotating wedges and expanding rings of contrast-reversing checkerboards. A flat map of the visual cortex was produced by the flattening procedure in MrVista [48] to visualize the spatial patterns and the functional activity of the ROIs.

#### 2.3.4. Statistical Analysis 

Given the limited sample size for each group in our study, we approached this paper as a “case report”, focusing on individual data visualization rather than cross-subject analysis. We followed a general rule of thumb for interpreting significance: if two error bars do not overlap, the difference is considered statistically significant, with an associated *p* value of less than 0.05 [49]. The correlation between V1 response and visual function improvements was tested using Spearman’s *rho* using the Real Statistics Resource Pack software in Excel (Copyright: 2013–2020, Charles Zaiontz. http://www.real-statistics.com (accessed on 31 January 2013)). 

## 3. Results

### 3.1. Visual Cortical Functions Pre- and Post-Perceptual Learning

**Figure 3:**
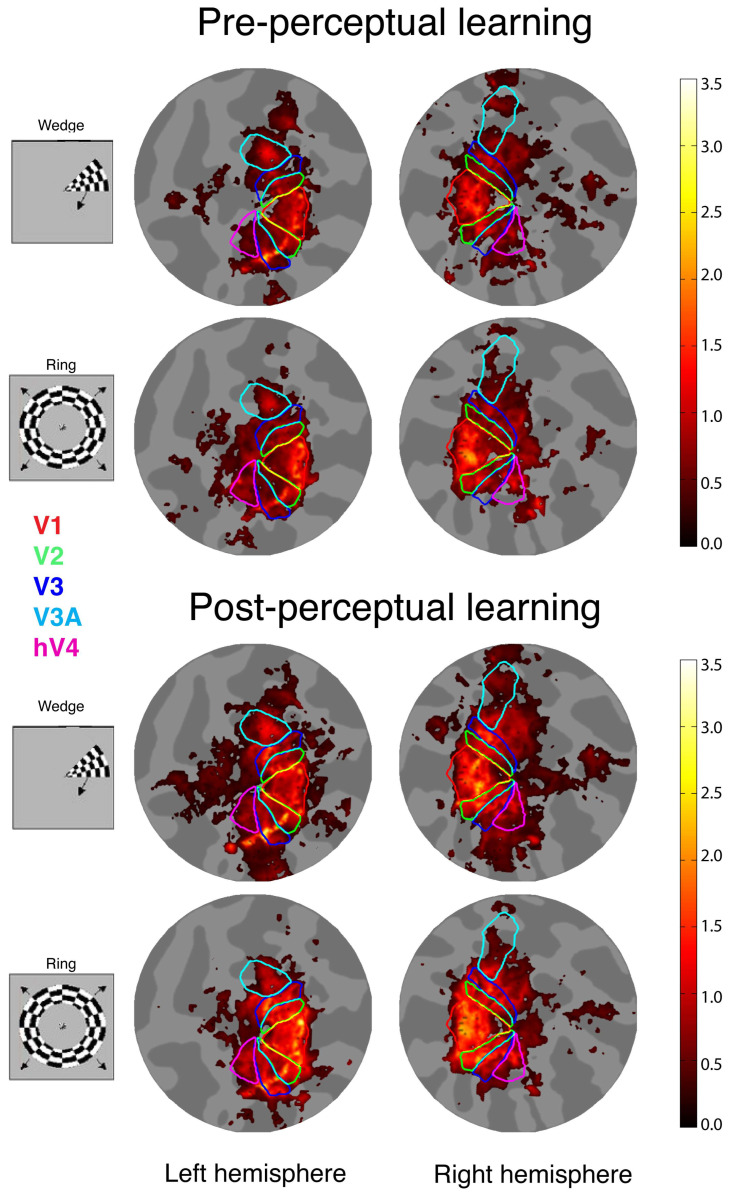
Sample flat maps of fMRI response amplitudes from one adult with amblyopia (Subject A1). Color outlines define ROIs, including V1, V2, V3, V3A, and hV4. Maps show stronger responses in ROIs, particularly in area V1, after perceptual learning compared to before learning.

We created flat maps of fMRI response amplitudes for each adult with amblyopia to compare visual cortical function changes before and after perceptual learning. In some amblyopes, particularly in the anisometropic subgroup, the flat maps showed stronger responses (brighter yellow) in the ROIs after perceptual learning, as demonstrated by a sample flat map from Subject A1, as shown in Figure 3. 

**Figure 4:**
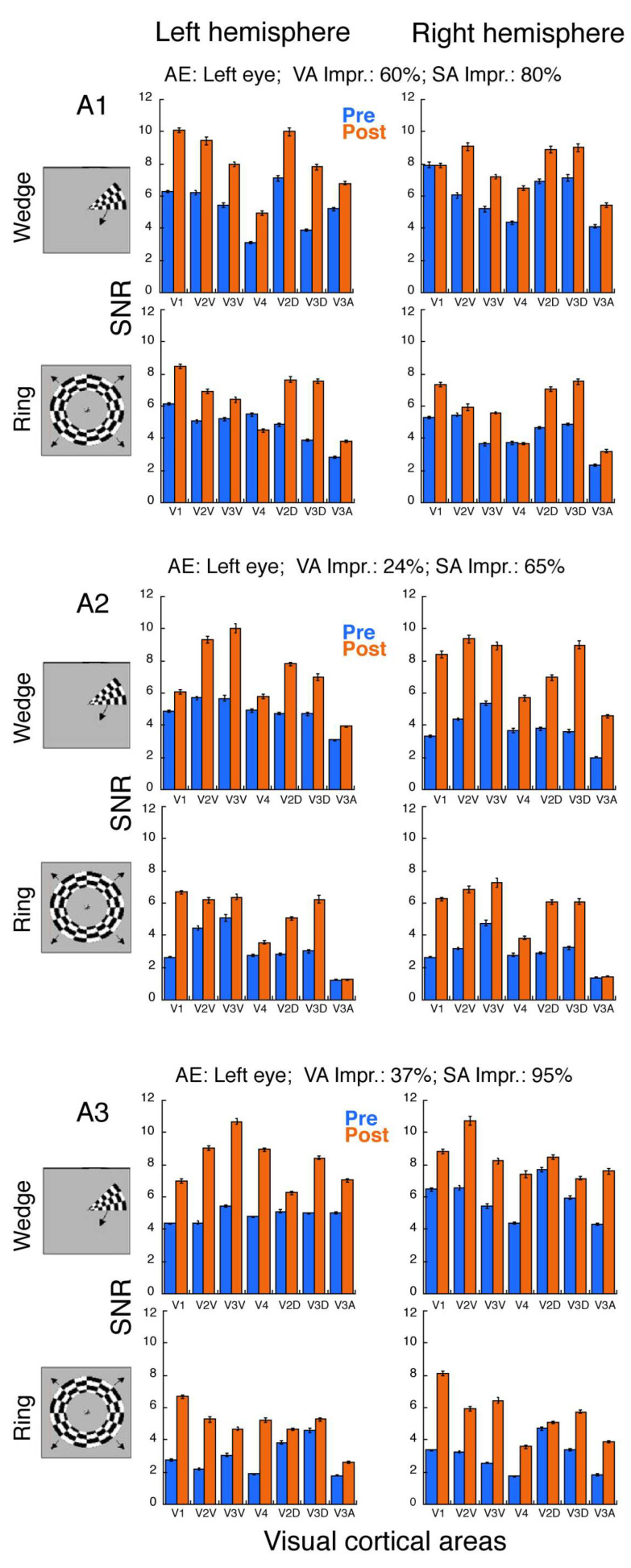
Signal-to-noise ratios (SNRs) from rotating wedges and expanding rings for each anisometropic amblyope. Perceptual learning resulted in increased responses across ROIs, including both ventral and dorsal processing are as, for all three anisometropic amblyopes. Error bars denote standard errors of means across voxels in each ROI. AE: amblyopic eye; VA: visual acuity; SA: stereoacuity; Impr.: improvement [(Post − Pre)/Pre × 100]. A1–A3: Subject ID in Table 1.

**Figure 5:**
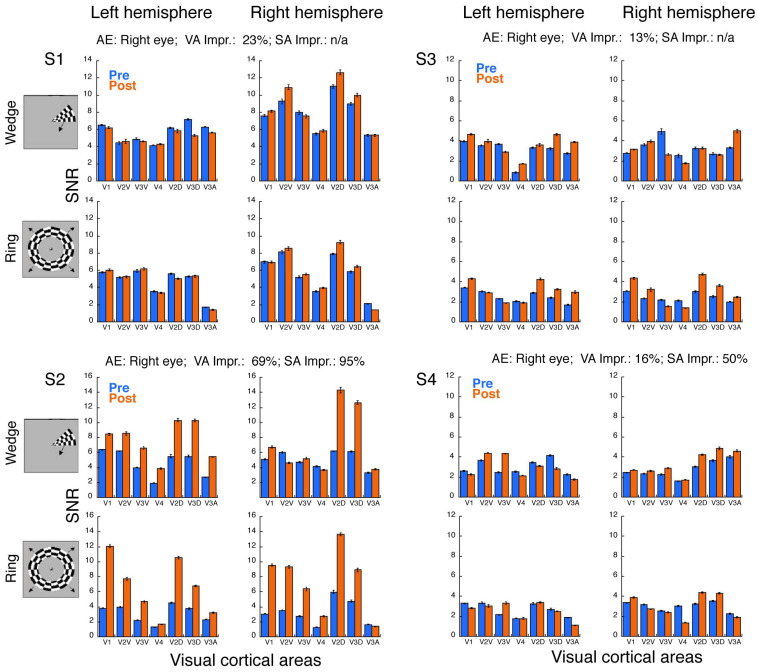
Signal-to-noise ratios (SNRs) of fMRI responses from rotating wedges and expanding rings for each strabismic amblyope. Except for Subject S2, strabismic amblyopes exhibited only slight increases in responses in certain ROIs after perceptual learning. However, Subject S2, who achieved improvements in visual acuity and stereoacuity, demonstrated robust increase in responses across all ROIs. Error bars denote standard errors of means across voxels in each ROI. AE: amblyopic eye; VA: visual acuity; SA: stereoacuity; Impr.: improvement [(Post − Pre)/Pre × 100]. S1–S4: Subject ID in Table 1.

Since two separate fMRI scans (pre- and post-training) were conducted in each subject, in order to control variability across two sessions, we extracted BOLD responses from gray matter and normalized the data by computing the signal-to-noise ratio (SNR) to demonstrate the effects of perceptual learning. The results are presented in Figure 4 for anisometropic amblyopia and Figure 5 for strabismic amblyopia. Overall, adults with anisometropic amblyopia (Figure 4) exhibited a robust increase in fMRI responses after learning across all ROIs. Notably, the error bars for the pre- and post-training data did not overlap, suggesting that the difference is likely statistically significant [49]. These anisometropic amblyopes gained an average improvement of 69% in visual acuity and 95% in stereoacuity. We will follow the same rule of thumb (whether the error bars for the pre- and post-training data overlap or not) to visualize Figure 5 for strabismic amblyopia. As demonstrated in Figure 5, except for Subject S2, most strabismic amblyopes showed little to no increase in fMRI responses across some ROIs. For instance, while Subjects S1, S3, and S4 displayed increased responses in V2D and V3D of the right hemisphere, Subjects S1 and S4 exhibited either no increase or only a weak increase in V1. The strabismic group (excluding Subject S2) gained an average visual acuity improvement of 17% through learning, with Subject S4 demonstrating a 50% improvement in stereoacuity, while Subjects S1 and S3 still had non-measurable stereoacuity post-learning. Interestingly, Subject S2, the patient with strabismic amblyopia who achieved significant improvements in both visual acuity (69%) and stereoacuity (95%), displayed robust increases in fMRI responses across all ROIs. These findings suggest that visual cortical function changes might be closely related to visual function improvements through learning, which we will explore further in the Results Section (see below).

Furthermore, a slight hemisphere bias was observed in cortical function improvement, though this bias varied depending on the individuals and the types of fMRI tests (Wedges vs. Rings). For instance, in the anisometropic group (Figure 4), Subjects A1 and A3 showed greater improvements in the left hemisphere during the wedge test, whereas A2 exhibited more improvements in the right hemisphere for both the wedge and ring tests. It is worth noting that the amblyopic eye for all three anisometropic subjects happened to be the left eye, indicating no correlation between hemisphere bias and whether the amblyopic eye was on the left or right side. In the strabismic group (Figure 5), S1 and S4 displayed relatively more improvements in the right hemisphere for both the wedge and ring tests, while S2 and S3 showed slightly more improvements in the left hemisphere during the wedge test. These findings further suggest that there is no clear correlation between hemisphere biases and whether amblyopia occurs in the left or right eye, as all four strabismic amblyopes had amblyopia in the right eye.

In addition, we observed cortical function improvements in both the ventral (V2V, V3V, and hV4) and dorsal (V2D, V3D, and V3A) pathways. However, no clear pattern of improvement differences between the ventral and dorsal processing pathways emerged across subjects. For example, while Subject A3 showed more improvement in the ventral pathway than in the dorsal pathway (e.g., V2V vs. V2D; V3V vs. V3D), other amblyopic subjects (e.g., A1, A2, S2, and S3) exhibited greater improvement in the dorsal pathway than in the ventral pathway. The extent of improvement appeared to depend on the hemispheres and the types of stimuli (wedge vs. radial). However, these observations require validation with a larger sample.

### 3.2. Visual Cortical Function Changes Link with Clinical Factors

Visual acuity and stereoacuity pre- and post-learning, adopted from Hou and Nicholas (2022) [13], are listed in Table 2, which were used for a correlation analysis as described below.

**Figure 6:**
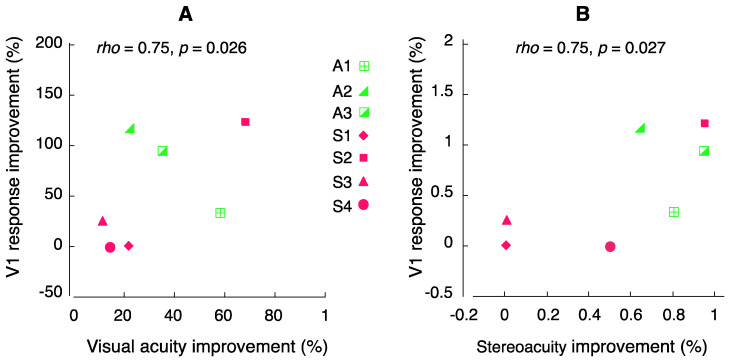
Correlation between V1 function improvement and visual function improvement after perceptual learning in adults with amblyopia. (**A**) Correlation between V1 response improvement and visual acuity improvement. (**B**) Correlation between V1 response improvement and stereoacuity improvement. Percent improvement was calculated as (Post − Pre)/Pre × 100. Correlation was tested using Spearman’s *rho*. Data show positive correlation, indicating that individuals with greater improvements in visual acuity and stereoacuity also gained greater V1 function improvement.

As pointed out earlier, our individual cases in Figure 4 and Figure 5 suggest that visual cortical function changes might be related to visual function improvements through learning. Thus, we quantified the relationship between the V1 function improvement and visual function improvement by learning. Neurons in V1 have the smallest receptive field size (i.e., the highest resolution), which correlates with visual acuity [50]. Therefore, it is meaningful to correlate visual function improvement with V1 function improvement. We took an average of V1 response improvements from the two hemispheres (left and right) and the two stimulus types (wedge and ring) for each subject and correlated with their visual acuity improvements and stereoacuity improvements in all seven amblyopes, as shown in Figure 6. Our results demonstrate a positive correlation between V1 function improvement and visual function improvement, including both visual acuity (Figure 6A) and stereoacuity (Figure 6B). This correlation is further confirmed by the observations from individual subjects in Figure 4 and Figure 5 by quantitative analysis, suggesting that individuals with greater improvements in visual acuity and stereoacuity also gained greater V1 function improvement.

## 4. Discussion

In this study, we explored whether perceptual learning using high-attention-demand tasks, which avoided contrast- or spatial frequency-based low-level processing tasks and required high-level cortical functions, induces changes in early visual cortical function in adults with amblyopia. Our case study showed that perceptual learning led to improvements in early cortical function, notably in areas V1, V2, V3, V3A, and hV4, in both the dorsal and ventral regions. These improvements were strongly associated with the etiology of amblyopia and the improvements in visual function through learning, with individuals experiencing greater visual function improvement also demonstrating more V1 functional gains.

### 4.1. Neural Correlates of Visual Function Recovery in Amblyopia Through Perceptual Learning

Over the past few decades, numerous studies have shown improvements in visual acuity and other visual functions in amblyopia through various approaches, including perceptual learning (see Tsirlin et al., 2015 [18], for a review). However, the neural correlates of visual function recovery through perceptual learning in amblyopia remain unclear, and there is still limited documentation in this area. Psychophysical studies in normal vision have shown that perceptual learning is highly specific to the task being trained. For example, improvements in stimulus orientation or spatial frequency [23,51], as well as position [23,52], do not transfer to non-trained tasks. Therefore, the findings of Zhai et al. (2013) [22], who reported increased activation in V1 after learning with cut-off spatial frequencies in individuals with anisometropic amblyopia, are expected. This is because learning tasks, such as those targeting “cut-off” spatial frequency, specifically involve V1 functions [27]. Interestingly, our study demonstrates that using high-attention-demand tasks, which engage IPS functions and high-level cortical process, also led to early cortical function improvements, such as in areas V1, V2, V3, V3A, and hV4, including both dorsal and ventral regions.

### 4.2. Evidence of Perceptual Learning Engaging Both Low- and High-Level Cortical Processes

Our training included three key factors (searching [40], counting [34], and attentional cueing [34]) and implemented selective visual attention in the tasks, thus incorporating “high attention demand”. These tasks required spatial attention and engaged high-level cortical functions, such as those found in the IPS [38,39,53]. The training stimuli ruled out low-level (i.e., V1) processing factors such as feature visibility due to low contrast/luminance or high spatial frequency (our tasks were counting the Gabors with two cycle/deg spatial frequency at above 25% contrast). Thus, we initially anticipated little to no increase in V1 activation. However, this was not the case. Despite the limited number of subjects, we observed a robust increase in V1 activation, especially in individuals with anisometropic amblyopia (Figure 4). There is limited documentation on this topic in the literature. In a behavioral study, Zhang et al. (2014) [54] demonstrated that perceptual learning in adults with amblyopia could be enabled to transfer completely to an orthogonal orientation, suggesting that amblyopic perceptual learning mainly results from high-level cognitive compensation. In our case, the searching and counting tasks represented high-level cortical functions (e.g., IPS as attention region [55]). Learning with such tasks led to low-level visual cortical function improvement in which the improvements in visual acuity and stereoacuity were significantly correlated with the magnitude of V1 function enhancement. Our findings provide evidence that perceptual learning engages both low- and high-level cortical processes.

It has been previously reported that attention enhances behavioral performance primarily by enabling the efficient selection and pooling of early sensory responses in the visual cortex [56]. The interaction between low-level local circuits (such as V1 neurons) and feedback connections from higher-order cortical areas likely plays a role in learning transfer [56,57] and perceptual learning mechanisms [20,21]. Our findings, obtained using high-attention-demand tasks in amblyopic training and conducting evaluations using fMRI, provide evidence supporting the view that the interaction between low-level local circuits (such as V1 neurons) and feedback connections from higher-order cortical areas likely plays a role in learning transfer and learning mechanisms.

## 5. Conclusions and Limitations

As with many perceptual learning studies, the need for multiple lab visits posed challenges, leading to a small sample size in our study. Consequently, we opted to present direct visualizations of the learning effects on the visual cortex for individual participants (Figure 4 and Figure 5) rather than averaged data across participants. Our findings provide proof-of-concept evidence that learning tasks engaging high-level cortical functions can lead to improvements in lower-level cortical areas, such as V1. The improvements in V1 function were strongly associated with the etiology of amblyopia (i.e., anisometropia vs. strabismus) and the degree of visual function improvement. Anisometropic amblyopes exhibited functional improvements across all regions of interest (V1, V2, V3, V3A, and hV4), along with gains in visual acuity and stereoacuity. In contrast, strabismic amblyopes only showed cortical function improvements in cases where significant visual function recovery was observed. These findings suggest that early interventions aimed at correcting eye alignment (e.g., strabismus surgery) may be critical for restoring both visual and cortical functions in strabismic amblyopia. However, whether there is a hemispheric bias or a ventral–dorsal bias in the learning effects remains to be explored in studies with larger samples.

## Figures and Tables

**Table 1:** Clinical details of the participants with amblyopia.

ID				^‡^ Visual Acuity (logMAR)		^‡^ Stereoacuity	Refractive Errors			
	Diagnosis	Age	Gender	Fellow Eye	Amblyopic Eye		Fellow Eye	Amblyopic Eye	Deviation	History
A1	A	52	F	0.04	0.50	100″	−1.75 + 1.00 × 10	−5.00 + 0.75 × 160	ortho	patching
A2	A	51	F	0.00	0.50	200″	−0.25	+3.00 + 0.50 × 90	ortho	patching
A3	A	50	F	−0.20	0.38	200″	−1.00 + 0.50 × 30	+4.5 + 0.50 × 150	ortho	patching
S1	S&A	59	M	−0.04	0.70	n/a	+0.75	−1.00 + 0.75 × 25	XT 14, L/R 4	surgery
S2	S&A	40	F	0.00	0.52	n/a	+3.25 + 2.00 × 170	Plano	XT 8	surgery
S3	S&A	66	F	−0.02	0.46	n/a	+1.25 + 1.00 × 105	+3.50 + 2.25 × 85	XT 8	surgery
S4	S&A	28	M	−0.09	0.62	400″	Plano	+100 + 0.50 × 90	ET 6	no patching

A: anisometropic amblyopia; S&A: mixed strabismus and anisometropia. M: male; F: female. Deviation near 33 cm with best optical correction is shown in prism diopters. XT: exotropia. ET: esotropia; L/R: left-eye hypertropia. n/a indicates participants who had non-measurable stereoacuity. ^‡^ indicates entrance level of logMAR acuity and stereoacuity. Adopted from Hou and Nicholas (2022) [13].

**Table 2:** Visual functions pre- and post-perceptual learning.

ID	Diagnosis	Age	Visual Acuity (logMAR) in AE			Stereoacuity		
			Pre	Post	Improv.	Pre	Post	Improv.
A1	A	52	0.50	0.20	0.60	100″	20″	0.80
A2	A	51	0.50	0.38	0.24	200″	70″	0.65
A3	A	50	0.38	0.24	0.37	200″	80″	0.95
S1	S&A	59	0.70	0.54	0.23	n/a	n/a	n/a
S2	S&A	40	0.52	0.16	0.69	n/a	200″	0.95
S3	S&A	66	0.46	0.40	0.13	n/a	n/a	n/a
S4	S&A	28	0.62	0.52	0.16	400″	200″	0.50

A: anisometropic amblyopia; S&A: mixed strabismus and anisometropia. AE: amblyopic eye; n/a: participants who had non-measurable stereoacuity. Improv.: improvement fraction [(Pre − Post)/Pre]. Adopted from Hou and Nicholas (2022) [13].

## Data Availability

The original contributions presented in this study are included in the article and also shared online (https://www.openicpsr.org/openicpsr/reviewPublish?resourcePath=/openicpsr/209336&type=project&tenant=openicpsr (accessed on 9/26/2024)). Further inquiries can be directed to the corresponding author.
